# Supplementary material for: Cosmic-void observations reconciled with primordial magnetogenesis
Source: Nat Commun. 2023 Nov 18;14:7523. doi: 10.1038/s41467-023-43258-3 (PMC10657398; doi:10.1038/s41467-023-43258-3)
Supplement: Supplementary file 1 — Supplementary Information [file 41467_2023_43258_MOESM1_ESM.pdf]

Cosmic-void observations reconciled with primordial magnetogenesis

David N. Hosking[1, 2, 3, 4, *] and Alexander A. Schekochihin[3, 5]

[1] *Oxford Astrophysics, Denys Wilkinson Building, Keble Road, Oxford OX1 3RH, UK*
[2] *Princeton Center for Theoretical Science, Princeton University, Princeton, NJ 08544, USA*
[3] *Merton College, Merton Street, Oxford, OX1 4JD, UK*
[4] *Gonville & Caius College, Trinity Street, Cambridge, CB2 1TA, UK*
[5] *The Rudolf Peierls Centre for Theoretical Physics, University of Oxford, Clarendon Laboratory, Parks Road, Oxford, OX1 3PU, UK*

(Dated: October 27, 2023)

SUPPLEMENTARY INFORMATION

A. Decay of helical PMFs

For completeness, here we provide the results for maximally helical fields that correspond to those presented in the main text for non-helical fields; these results are relevant for magnetogenesis mechanisms that are capable of parity violation. The decay of such fields conserves the net magnetic helicity, resulting in the self-similar scaling

$$\langle h \rangle = \tilde{B}^2 \lambda_B \sim \text{const.} \tag{1}$$

As in the non-helical case, the decay proceeds on reconnection timescales [1]; the possible decay regimes are the same as those described in the main text. Under Equation (1), the PMF evolution in the $(\tilde{B}, \lambda_B)$ plane is parallel to Equation (1) of the main text [see Supplementary Fig. 1]. Thus, any field satisfying

$$\tilde{B}(t_*) \gtrsim 10^{-17}\,\mathrm{G} \left[\frac{\lambda_B(t_*)}{1\,\mathrm{Mpc}}\right]^{-1/2} \tag{2}$$

will satisfy the observational constraint [Equation (1) of the main text] at recombination, as is well known (see, e.g., [2, 3]).

The locus of present-day PMF states for decays that occur on the reconnection timescales explained in the main text is shown by the blue-gold line in Supplementary Fig. (1). Analogously to $I_{\mathbf{L},\,\max}$ and $I_{H,\,\max}$ in the main text, we denote the largest value of the mean magnetic-helicity density $\langle h \rangle$ that is consistent with EWPT magnetogenesis by $\langle h \rangle_{\max}$ [this corresponds to $\tilde{\rho}_B(t_*) = \tilde{\rho}_\gamma(t_*)$ and $\lambda_B(t_*) = r_H(t_*)$]. For $\langle h \rangle \lesssim 10^{-15} \langle h \rangle_{\max}$, the decay of PMFs terminates on line (i) in Supplementary Fig. 1, which corresponds to Equation (14) of the main text with $\mathrm{Pm} \sim \mathrm{Pm}_{\mathrm{Sp}}$ [Equation (37) of the main text]. For $10^{-15}\langle h \rangle_{\max} \lesssim \langle h \rangle \lesssim 10^{-11} \langle h \rangle_{\max}$, the decay of PMFs terminates on line (ii) [Equation (45) of the main text], which corresponds to Equation (14) of the main text with $\mathrm{Pm} = (r_L/\lambda_{\mathrm{mfp}})^2 \mathrm{Pm}_{\mathrm{Sp}}$. For $10^{-7}\langle h \rangle_{\max} \lesssim \langle h \rangle \lesssim 10^{-5} \langle h \rangle_{\max}$, decays terminate at $\tilde{B} \sim 10^{-11}$, G, which corresponds to $\delta_c \sim \lambda_{\mathrm{mfp}}$, as explained in the main text. Finally, decays are radiation-drag limited for $\langle h \rangle \gtrsim 10^{-5} \langle h \rangle_{\max}$, and therefore terminate on line (iv) [Equation (55) of the main text]. We note that, for $\langle h \rangle \lesssim 10^{-5} \langle h \rangle_{\max}$, the role of magnetic reconnection in determining the decay timescale implies significantly stronger relic fields than would be expected under the decay physics envisaged by [4], i.e., Alfvénic [Equation (9) of the main text; line (v)] or radiation-drag-limited [line (iv)] decay.

As in the main text, we also indicate by a red-gold line the locus of present-day PMF states if $\mathrm{Pm} \lesssim 1$ (due to plasma microinstabilities) for $\tilde{B} \gtrsim \tilde{B}_{\mathrm{iso}}$.

---

* dhosking@princeton.edu

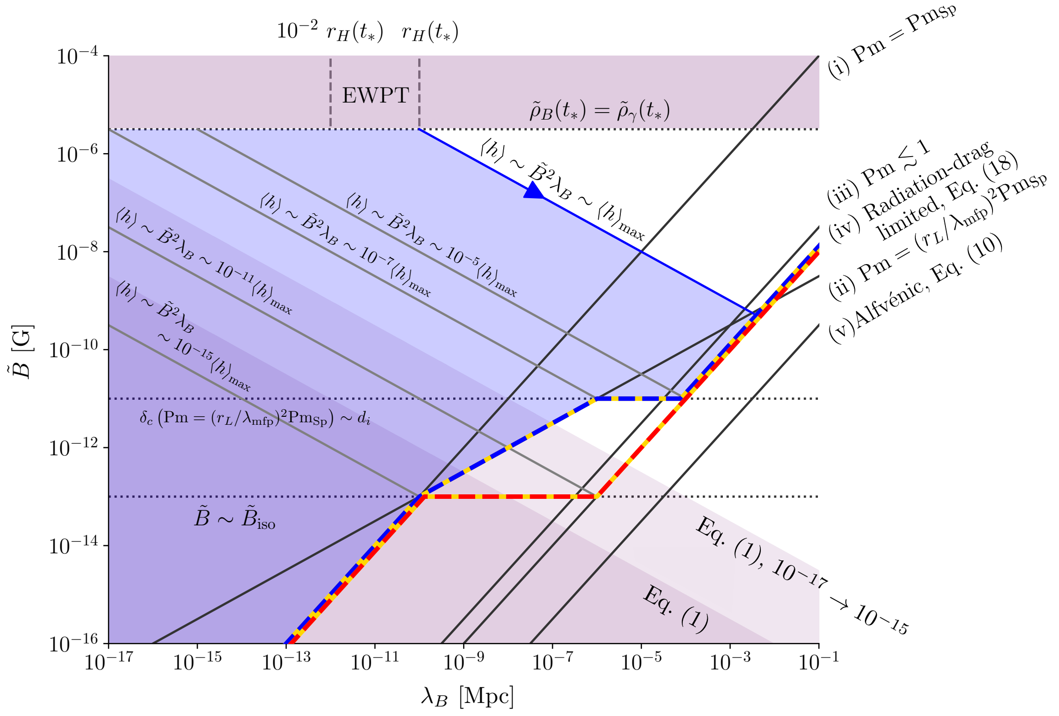

Figure 1. **Evolution of a maximally helical PMF.**
As in Fig. 3 of the main text, purple regions denote values of $\tilde{B}$ and λ_B excluded on physical or observational grounds [Equation (1) of the main text]. Under decays that conserve $\langle h\rangle = \langle \mathbf{A}\cdot\mathbf{B}\rangle$ [Equation (1)], $\tilde{B}$ and λ_B evolve along lines parallel to the ones shown in blue. The predicted values of modern-day $\tilde{B}$ and λ_B are given by the intersection of these lines with Equation (8) of the main text evaluated at recombination [represented by lines (i-v), which are derived in Methods], with τ the prevailing decay timescale. The blue-gold line shows the locus of possible present-day states resulting from reconnection-controlled decays on the timescales explained in the main text, assuming that the microscopic viscosity of the primordial plasma was controlled by collisions between protons. The effective value of Pm in Equation (14) of the main text might have been heavily suppressed when $\tilde{B} > \tilde{B}_{\rm iso}$ if viscosity were then instead governed by plasma microinstabilities — the red-gold line shows the locus of modern-day states corresponding to the extreme choice of Pm $\lesssim 1$ for $\tilde{B} > \tilde{B}_{\rm iso}$.

B. Decay of non-helical magnetic fields with $I_H = 0$

As explained in the main text, the invariance of I_H follows from the conservation of the fluctuation level of magnetic helicity. While we view fluctuations in magnetic helicity to be a generic feature of real MHD turbulence (and all extant numerical work on this subject, e.g., [4–13], has employed initial conditions with helicity fluctuations exclusively), it is nonetheless possible to construct artificial field configurations for which the helicity of each magnetic structure vanishes — this will be the case if they have no twists and do not interlink. Strictly, therefore, the possibility that PMFs might have been generated without helicity fluctuations cannot be ruled out.

A priori, it appears that this kind of field might relax in a fundamentally different manner to the one described in the main text. This was the view that we expressed in Ref. [1]: there, we suggested that fields with $I_H = 0$ might decay subject to the conservation of invariants associated with the velocity, rather than the magnetic, field. This is because individually non-helical structures (unlike helical ones) can relax under entirely flux-frozen dynamics, by driving flows with $\tilde{u} \sim \tilde{B}$ (a process sometimes called kinetic diffusion [2]). Plausibly, the decay of those flows would respect the invariance of the hydrodynamic Loitsyansky integral,

$$I_{\mathbf{L}} \equiv -\int \mathrm{d}^3\mathbf{r}\, r^2 \langle \tilde{\mathbf{u}}(\mathbf{x})\cdot\tilde{\mathbf{u}}(\mathbf{x}+\mathbf{r})\rangle, \quad (3)$$

which encodes the conservation of angular momentum $\mathbf{L} = \mathbf{x} \times \mathbf{u}$ [14] (in the same fluctuating manner as I_H encodes helicity conservation [1]). $I_{\mathbf{L}}$ is related to the small-k asymptotic of the kinetic-energy spectrum, $\mathcal{E}_K(k)$, of isotropic turbulence without long-range spatial correlations by $\mathcal{E}_K(k \to 0) = I_{\mathbf{L}} k^4/24\pi^2 + O(k^6)$ [15]. Denoting the characteristic size and scale of the velocity field by $\tilde{u}$ and λ_u respectively, $I_{\mathbf{L}} \sim \tilde{u}^2 \lambda_u^5$. Conservation of $I_{\mathbf{L}}$ therefore implies $\tilde{u}^2 \lambda_u^5 \sim$ const. This suggests that $\tilde{B}^2 \lambda_B^5 \sim$ const also, if $\tilde{u} \sim \tilde{B}$ and $\lambda_B \sim \lambda_u$, which seems reasonable for, e.g., a magnetic field maintained by the dynamo effect (we note that the dynamo effect in a decaying velocity field has been studied by [11]). This returns us to Equation (6) of the main text, i.e., to the same prediction that was shown to be inconsistent with the observational constraints by Ref. [16].

On the other hand, if the magnetic field were maintained by dynamo, then it seems unlikely that $I_H = 0$ would be maintained. This is because random helicity fluctuations could be generated freely at resistive scales (as the Lundquist number is order unity there), where dynamo primarily generates magnetic field (at least in its kinematic stage) [17]. Thus, I_H could become non-zero, although it would not need to be conserved if magnetic energy remained concentrated at the resistive scales. If, however, the dynamo-replenished magnetic fields later transferred to larger scales and saturated with $\lambda_B \sim \lambda_u$, as supposed above, while still having helicity fluctuations, then I_H would become invariant, because the integral scale of the magnetic field would be much larger than the resistive scale. This would push us back to the scaling $I_H \sim \tilde{B}^4 \lambda_B^5 \sim$ const [Equation (13) of the main text]. Moreover, we conjecture that the size of the conserved product $\tilde{B}^4 \lambda_B^5$ would be of the same order as its value for the initial field with $I_H = 0$, because memory of its $\tilde{B}$ and λ_B would be retained by the velocity field. In Supplementary Fig. 2, we present results from a numerical simulation (Simulation A) designed to assess these arguments. We initialise a large number of untwisted, non-interlinking magnetic-flux loops (otherwise distributed in a random, statistically isotropic way) in a periodic simulation domain [see Supplementary Fig. 2(a)]. At $t = 0$, $I_H = 0$ because the loops each have zero magnetic helicity. For the purpose of comparison, we also present a second simulation (Simulation B) with the same setup but without the non-interlinking condition, instead starting with many loops superimposed on top of each other. This field has complex initial topology, as Supplementary Fig. 2(b) indicates, but no net helicity, in the sense that $\langle H_V \rangle / V \ll \tilde{B}^2 \lambda_B$ for all V. However, unlike the field in Simulation A, it has $I_H \neq 0$, because superimposing loops creates linkages in the magnetic field.

Supplementary Figs. 2(c-d) show the evolution of energy spectra for the two simulations. In Simulation A, unlike in Simulation B, there is an immediate and rapid decay of the magnetic energy as the loops contract and drive flows. There is a corresponding decay of the large-scale (low-k) spectral tail, which demonstrates the non-invariance of $I_{\mathbf{L}_M}$. The newly generated kinetic energy is comparable in magnitude to the initial magnetic energy [see Supplementary Fig. 2(i)], and its spectrum peaks close to the initial peak of the magnetic-energy spectrum. At larger scales, it exhibits a power law close to $\mathcal{E}(k) \propto k^2$, suggesting that it is a 'Saffman turbulence' — roughly speaking, eddies are translational rather than rotational [15]. However, we note that each flux tube must individually relax in a momentum-conserving manner, so it is unlikely that the relaxation could generate true Saffman turbulence, which has a stochastic momentum distribution. Instead, it is likely that the momentum distribution is "quasi-random", in the sense described by Ref. [18] — in an arbitrarily large simulation domain, one would find that the $\mathcal{E}_K(k) \propto k^2$ spectrum transitions to $\mathcal{E}_K(k) \propto k^4$ at sufficiently large scales. Similarly, the large-scale spectrum of the magnetic energy appears to be somewhat shallower than $\mathcal{E}_M(k) \propto k^4$ — we think that this too is an effect of the finite size of the simulation domain. The contraction of the loops leaves magnetic energy concentrated at small (resistive) scales, where it can be refuelled by the dynamo effect associated with the newly generated flows.

As we anticipated above, the resistive-scale magnetic field has random fluctuations in magnetic helicity: this is shown explicitly in Supplementary Fig. 2(e) where, for volumes V taken to be spheres of radius R, we plot $\langle H_V^2 \rangle / V$ vs. R at regular intervals in time (the average is taken over a large sample of spheres with centres throughout the simulation box). While $\langle H_V^2 \rangle \propto V^{2/3}$ at $t = 0$ (not shown) because H_V is dominated by random surface contributions at this time, this scaling is replaced by $\langle H_V^2 \rangle \propto V$ as soon as turbulence develops, indicating $I_H \neq 0$ [see Equation (12) of the main text]. Though I_H, which is the value of $\langle H_V^2 \rangle / V$ in the flat part of the curves in Supplementary Figs. 2(e-f), decays by around an order of magnitude during the first few eddy-turnover times, Supplementary Fig. 2(e) shows

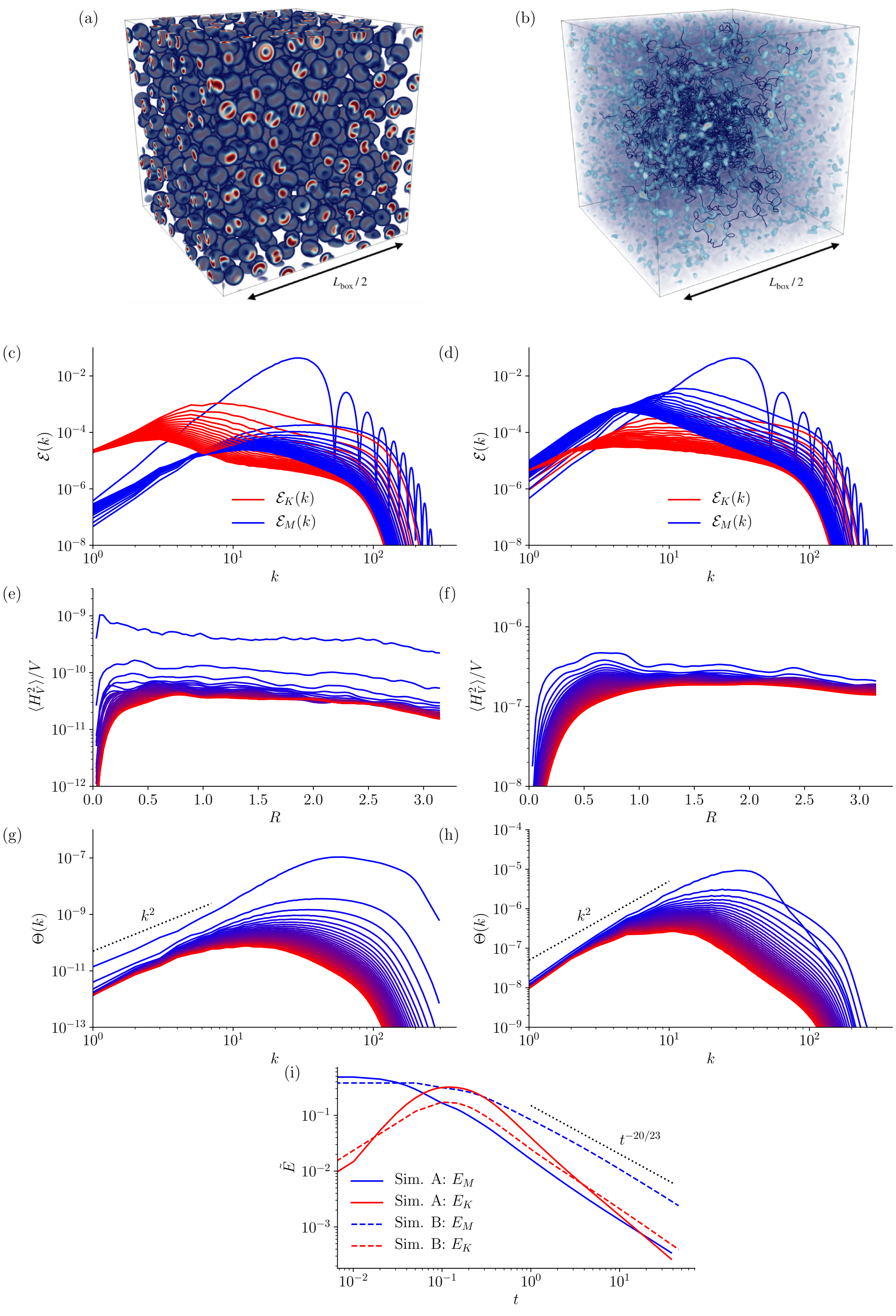

Figure 2. **Simulations of MHD turbulence decaying from a magnetically dominated state.** Left-hand plots are for Simulation A, which had $I_H = 0$ initially; right-hand plots are for Simulation B, which had $I_H \neq 0$ initially. Panels (a-b) show a 3D plot of the magnetic-energy distribution at the initial time, in a volume 1/8 the size of the simulation domain (the box size $L_{\rm box} = 2\pi$ in code units); panels (c-d) show energy spectra (kinetic in red, magnetic in blue), plotted at intervals of 2.0 between $t = 0$ and $t = 38.0$ (time and other quantities are measured in code units based on normalising the box size and the mean-square magnetic field to 2π and 1, respectively, so that one time unit is equal to the initial Alfvén crossing time of the box); panels (e-f) show $\langle H_V^2\rangle/V$ (computed as an average over many spheres of radius R and volume V, distributed throughout the simulation domain) vs. R, plotted at intervals of 1.0 between $t = 0.25$ (blue) and $t = 38.25$ (red); panels (g-h) show the helicity-variance spectrum $\Theta(k)$, at the same times as for (e-f); panel (i) shows the evolution of magnetic energy E_M and kinetic energy E_K for each simulation as functions of time, with the theoretical prediction for the decay on the slow-reconnection timescale, $E_M \propto t^{-20/23}$, given for reference [this follows from Equation (14) of the main text, generalised appropriately for hyper-dissipation, with $S < S_c$ — see [1]]. Source data for this figure are provided as a Source Data file.

that its decay ceases after that. This is consistent with our suggestion above that I_H should become constant when the dynamo saturates, due to migration of the helicity-containing scale towards the flow scale λ_u. This interpretation is supported by the evolution of the helicity-variance spectrum $\Theta(k)$ [see Supplementary Fig. 2(g)], which encodes the characteristic size of helicity fluctuations at each scale (note that $\Theta(k)$ is not the same as the helicity spectrum, which is close to zero for all k for both simulations, as the field is non-helical at all scales). In Simulation A, $\Theta(k)$ is concentrated around the dissipation scale at early times (though after the decay of the magnetic loops), but later moves to larger scales. I_H [which is proportional to the coefficient of k^2 in the $\Theta(k \to 0) \propto k^2$ asymptotic [1]] ceases to decay once the peak of $\Theta(k)$ is moderately separated from the dissipation scales.

The value of I_H ultimately attained by the magnetic field in Simulation A is smaller than the one in Simulation B by a factor of around 10^4. This appears to contradict our conjecture that dynamo should generate I_H of the same size as $\tilde{B}^4\lambda_B^5$ at the initial time. On the other hand, we note that (i) this factor may well be smaller for a simulation at larger resolution and larger Prandtl number (recent work has shown that extremely large resolutions are required to probe the asymptotic nature of the large-Pm dynamo [19]), and that (ii) the strong scaling of I_H with $\tilde{B}$ and λ_B means that even a factor-10^4 reduction in I_H corresponds only to a factor-10 reduction in $\tilde{B}$ or λ_B. This means that a PMF generated with $I_H = 0$ would migrate only a relatively short distance on the $(\tilde{B}, \lambda_B)$ plane (Fig. 3 of the main text) before settling to decay with $\tilde{B}^4\lambda_B^5 \sim \text{const}$.

To summarise, there appear to be both theoretical and numerical reasons to believe that a PMF generated with $I_H(t_*) = 0$ at the initial time t_* would, via an initial period of rapid decay and subsequent regeneration via dynamo, develop $I_H \sim \tilde{B}(t_*)^4\lambda_B(t_*)^5 \sim \text{const}$. At later times, a magnetically dominated state would likely be re-established because the flows will drive Alfvénic turbulence, which cascades to small scales and is dissipated by viscosity (which may be large, if associated with neutrinos or photons), while background "quasi-force-free" magnetic fields persist, decaying only on the magnetic-reconnection timescale, as described in the main text. There is some evidence of this in Supplementary Fig. 2(i), which shows that magnetic energy becomes larger than kinetic in Simulation A at late times.

C. The effect of the large-scale spectral slope: coexistence of flux and helicity invariants

In this paper, we have contrasted our theory of I_H-conserving PMF decay with the previously accepted theory based on "selective decay of small-scale structure", i.e., the invariance of the large-scale asymptotic of the magnetic-energy spectrum. One success of our theory is that it explains the inverse-transfer effect observed in simulations of magnetic fields initialised with $\mathcal{E}_M(k \to 0) \propto k^4$ ([10, 20]; see main text); this effect is manifestly not compatible with selective decay. On the other hand, Ref. [12] observe that inverse transfer is *not* present in simulations that are initialised with sufficiently shallow large-scale spectra [namely, with $\mathcal{E}_M(k \to 0, t = 0) \propto k^n$, where $n < 3$]. Instead, they find that the $k \to 0$ asymptotic of $\mathcal{E}_M(k)$ is preserved. This result raises questions of whether a "selective-decay-like" principle might be at work in such decays, and what its effect might be on the laws for the decay of energy and growth of the integral scale. In this Section, we explain the invariance of this k^n asymptotic as a consequence of the conservation of magnetic flux, but also argue that, beyond an initial transient, flux conservation does not affect the decay laws if $n > 3/2$ (as is the case in all models of EWPT magnetogenesis of which we are aware). It is therefore not necessary to know the precise value of n to compute the present-day properties of EGMFs under the relic-field hypothesis — the theory presented in the main text is valid independently of it.

1. *Invariance of the large-scale spectral asymptotic for $n \leq 3$*

In general, the large-scale spectral asymptotic is frozen in time when the coefficient of k^n in $\mathcal{E}_M(k \to 0)$ is proportional to some statistical invariant. As explained in the main text, this is not the case when correlations in $\mathbf{B}$ decay rapidly with distance, because then $\mathcal{E}_M(k \to 0) \propto I_{\mathbf{L}_M} k^4$ [Equation (5) of the main text] where $I_{\mathbf{L}_M} \neq \text{const}$.

However, for $n \leq 3$, it turns out that the coefficient of k^n is proportional to an invariant that is related to the conservation of magnetic flux. Physically, this invariant encodes the fact that, over sufficiently large volumes, local fluctuations in magnetic flux may sum to a non-zero net fluctuation level, which must be conserved as the field decays. Spatial correlations must be long (and hence spectra must be shallow) for the fluctuation level to be non-zero, because $\nabla\cdot\mathbf{B} = 0$ means that magnetic structures without sufficiently strong far-field components have net zero flux. The relevant measure of correlation strength is the large-r asymptotic of the magnetic field's longitudinal correlation function, $\chi_B(r) \equiv \langle B_r(\mathbf{x}) B_r(\mathbf{x}+\mathbf{r})\rangle / \langle B_r^2\rangle$, where $B_r = \mathbf{B}\cdot\mathbf{r}/r$. The argument is particularly transparent if $\chi_B(r\to\infty) \propto r^{-3}$ (as, for example, would be the case for a superposition of many randomly positioned and oriented magnetic dipoles), as then it can be shown that

$$\mathcal{E}_M(k\to 0) = \frac{I_{\mathbf{B}} k^2}{4\pi^2}, \tag{4}$$

where

$$I_{\mathbf{B}} \equiv \int \mathrm{d}^3\mathbf{r} \langle \tilde{\mathbf{B}}(\mathbf{x})\cdot\tilde{\mathbf{B}}(\mathbf{x}+\mathbf{r})\rangle = \lim_{V\to\infty} \frac{1}{V} \left\langle \left(\int_V \mathrm{d}^3\mathbf{x}\, \tilde{\mathbf{B}} \right)^2 \right\rangle \equiv \lim_{V\to\infty} \frac{\langle \tilde{B}_V^2 \rangle}{V} \tag{5}$$

is the Saffman flux invariant [1]. The invariance of $I_{\mathbf{B}}$ encodes conservation of the fluctuation level of magnetic flux in the same manner as the invariance of I_H does for magnetic helicity. More generally, if $\mathcal{E}_M(k\to 0) = Ck^n$ with $n > -1$, then it can be shown that χ_B satisfies

$$\chi_B(r\to\infty) \begin{cases} \leq O(r^{-1-n}) & \text{if } n = 2m,\ m = 2,3,4,\ldots; \\ = f_n C r^{-1-n} & \text{otherwise,} \end{cases} \tag{6}$$

where f_n is a numerical coefficient that depends only on n. Furthermore, it can also be shown that

$$\lim_{R\to\infty} \langle \tilde{B}_V^2 \rangle \begin{cases} \propto R^2 & \text{if } n > 3, \\ = g_n C R^2 \ln R & \text{if } n = 3, \\ = g_n C R^{5-n} & \text{if } -1 < n < 3, \end{cases} \tag{7}$$

where g_n is a different numerical coefficient dependent only on n, and R is the radius of a spherical control volume V. These results are straightforward analogues of ones that we derived for the kinetic-energy spectrum of hydrodynamic turbulence in Ref. [18]. The rate of change of $\langle \tilde{B}_V^2 \rangle$ due to the advection of flux through the surface of V scales as

$$\frac{\mathrm{d}}{\mathrm{d}t}\langle \tilde{B}_V^2 \rangle \propto V^{2/3} \propto R^2 \implies \frac{\mathrm{d}}{\mathrm{d}t} \log\langle \tilde{B}_V^2 \rangle \propto \begin{cases} 1 & \text{if } n > 3, \\ 1/\ln R & \text{if } n = 3, \\ R^{n-3} & \text{if } -1 < n < 3, \end{cases} \tag{8}$$

so the timescale associated with changes in $\langle \tilde{B}_V^2 \rangle$ is an increasing function of R for $n \leq 3$. This means that the decay is constrained by the conservation of magnetic flux via

$$\lim_{R\to\infty} \frac{\langle \tilde{B}_V^2 \rangle}{R^{5-n}} = \text{const} = g_n C. \tag{9}$$

Equation (9) shows that $C = \text{const}$ for $n \leq 3$, which explains the invariance of $\mathcal{E}_M(k\to 0)$ observed by Ref. [12].

2. *Conservation of magnetic flux does not affect the decay laws for $\tilde{B}$ and λ_B*

We now turn to the effect that the need to satisfy the new constraint (9) has on the decay laws. A fully self-similar decay satisfying Equation (9) would have

$$\lim_{R\to\infty} \frac{\langle \tilde{B}_V^2 \rangle}{R^{5-n}} \sim \tilde{B}^2 \lambda_B^{1+n} \sim \text{const}, \tag{10}$$

which is the selective-decay scaling considered by [4]. However, Equation (10) cannot describe the true evolution as it is inconsistent with the invariance of I_H, as we now explain. While, in principle, I_H can be small compared to $\tilde{B}^4\lambda_B^5$ (see Section B of the Supplementary Information), it cannot be much larger than this: $I_H \sim \tilde{B}^4\lambda_B^5$ corresponds to magnetic fields that are locally maximally helical. Therefore, adopting Equation (10), we can write

$$I_H \lesssim B^4 \lambda_B^5 \sim \tilde{B}^{2(2n-3)/(n+1)}. \tag{11}$$

Assuming that $n > 3/2$, Equation (11) requires I_H to be smaller than a decreasing function of time, which contradicts its invariance.

On the other hand, the scaling $I_H \sim \tilde{B}^4\lambda_B^5 \sim$ const [Equation (13) of the main text] is *not* incompatible with Equation (9), as, under this scaling,

$$\lim_{R\to\infty} \frac{\langle \tilde{B}_V^2 \rangle}{R^{5-n}} \lesssim \tilde{B}^2 \lambda_B^{1+n} \sim \tilde{B}^{2(3-2n)/5}. \tag{12}$$

Again assuming that $n > 3/2$, Equation (12) only requires $\lim_{R\to\infty}\langle \tilde{B}_V^2 \rangle / R^{5-n}$ to be smaller than an increasing function of time, which does not contradict its conservation. We conclude that while the selective-decay scaling (10) is ruled out by I_H conservation, the converse is not true: Equation (13) of the main text is compatible with the conservation of the magnetic-flux fluctuation level, and thus with the invariance of the large-scale spectral asymptotic. We therefore expect Equation (13) of the main text to hold regardless of the value of n (although for $n < 4$, some transient order-unity variation in $\tilde{B}^4\lambda_B^5$ should be expected as a result of departures from self-similarity; see below).

That conservation of I_H should provide the relevant constraint even in the presence of magnetic-flux fluctuations is also reasonable physically. Under Equation (13) of the main text, Equations (9) and (12) imply that the expectation value of the squared magnetic flux contained within the volume V decreases relative to its "maximal" value of $\tilde{B}^2\lambda_B^{1+n}R^{5-n}$. This makes sense: while there is a dynamical tendency for magnetic fields to favour locally maximally helical states [21, 22] (meaning that we expect $I_H \sim \tilde{B}^4\lambda_B^5$), there is no physical reason that that they should maintain states of maximal magnetic flux (in the sense that $\lim_{R\to\infty}\langle \tilde{B}_V^2 \rangle / R^{5-n} \sim \tilde{B}^2\lambda_B^{1+n}$).

A schematic of the evolution of the magnetic-energy spectrum decaying in a manner that satisfies both (9) and Equation (13) of the main text is shown in Supplementary Fig. 3. Under Equation (13) of the main text, the spectral peak at $k \sim 1/\lambda_B$ grows relative to the position it would occupy under selective decay — i.e., there is an inverse transfer — nonetheless, the $k \to 0$ asymptotic is preserved. This leads to the development of a spectral knee at $k = k_c$, where k_c^{-1} is the minimal scale for the applicability of $\langle \tilde{B}_V^2 \rangle \propto R^{5-n}$. Because coalescence of structures via magnetic reconnection is a local process, we argue that it should not generate correlations on scales much larger than λ_B. This means that the spectrum between k_c and λ_B^{-1} should be proportional to k^4 [cf. Equation (5) of the main text; see [18] for discussion of the correspondence between long-range correlations and broken-power-law spectra].

The size of k_c can be estimated by equating the invariant asymptotic with the growing k^4 component of the spectrum, which on dimensional grounds is of size $\sim \tilde{B}^2\lambda_B^5 k^4$:

$$B_0^2 L_0 (kL_0)^n \sim \tilde{B}^2 \lambda_B^5 k^4. \tag{13}$$

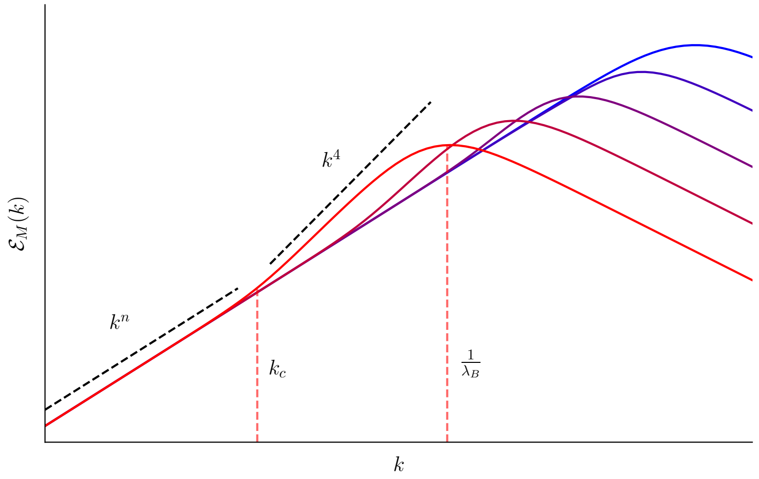

Figure 3. **Schematic of the evolution of $\mathcal{E}_M(t,k)$ for $\mathcal{E}_M(t=0,k\to 0)\propto k^n$.**
For $n \leq 3$, the $k \to 0$ asymptotic of $\mathcal{E}_M(t,k)$ is preserved as the turbulence decays; this is a consequence of magnetic-flux conservation. Nonetheless, the inverse-transfer effect persists, though it only occurs for $k > k_c(t)$, where k_c is given by Equation (14). The progression of time is from blue to red in this figure.

From $\tilde{B}^4\lambda_B^5 \sim$ const, we have

$$k_c \sim \frac{1}{\lambda_B}\left[\frac{\lambda_B}{\lambda_B(0)}\right]^{-\frac{2n-3}{2(4-n)}} \sim \frac{1}{\lambda_B}\left[\frac{\tilde{B}}{\tilde{B}(0)}\right]^{\frac{2(2n-3)}{5(4-n)}}, \tag{14}$$

a decreasing function of time.

The evolution of the magnetic-energy spectrum depicted in Supplementary Fig. 3 is manifestly not self-similar. As a result, transient order-unity changes in $\tilde{B}^4\lambda_B^5$ and the decay timescale as a function of $\tilde{B}$ and λ_B should be expected at early times. On the other hand, the decay does become approximately self-similar at late times, when $k_c \ll 1/\lambda_B$, so any deviation from the theory proposed in the main text becomes small as t becomes large. Finally, we acknowledge that, while we expect the evolution depicted in Supplementary Fig. 3 to be valid for any initial spectrum with $n > 3/2$, Ref. [12] do not observe the formation of a "k^4 bulge" in their simulation with $n = 2$. We believe this to be a result of insufficient scale separation in that simulation: with $n = 2$, Equation (14) implies $k_c\lambda_B \sim [\lambda_B/\lambda_B(0)]^{-1/4}$, so with $\lambda_B/\lambda_B(0) \simeq 10$ (see Fig. 16 of Ref. [12]), $k_c\lambda_B \simeq 0.6$. It is therefore not surprising that these scales cannot be distinguished.

[1] D. N. Hosking and A. A. Schekochihin, Reconnection-controlled decay of magnetohydrodynamic turbulence and the role of invariants, Phys. Rev. X **11**, 041005 (2021).
[2] R. Durrer and A. Neronov, Cosmological magnetic fields: their generation, evolution and observation, Astron. Astrophys. Rev. **21**, 62 (2013).
[3] K. Subramanian, The origin, evolution and signatures of primordial magnetic fields, Rep. Prog. Phys. **79**, 076901 (2016).
[4] R. Banerjee and K. Jedamzik, Evolution of cosmic magnetic fields: From the very early Universe, to recombination, to the present, Phys. Rev. D **70**, 123003 (2004).
[5] D. Biskamp and W.-C. Müller, Decay laws for three-dimensional magnetohydrodynamic turbulence, Phys. Rev. Lett. **83**, 2195 (1999).
[6] W.-C. Müller and D. Biskamp, Scaling properties of three-dimensional magnetohydrodynamic turbulence, Phys. Rev. Lett. **84**, 475 (2000).

[7] M. Christensson, M. Hindmarsh, and A. Brandenburg, Inverse cascade in decaying three-dimensional magnetohydrodynamic turbulence, Phys. Rev. E **64**, 056405 (2001).

[8] P. Frick and R. Stepanov, Long-term free decay of MHD turbulence, Europhys. Lett. **92**, 34007 (2010).

[9] A. Berera and M. Linkmann, Magnetic helicity and the evolution of decaying magnetohydrodynamic turbulence, Phys. Rev. E **90**, 041003 (2014).

[10] A. Brandenburg, T. Kahniashvili, and A. G. Tevzadze, Nonhelical inverse transfer of a decaying turbulent magnetic field, Phys. Rev. Lett. **114**, 075001 (2015).

[11] A. Brandenburg and T. Kahniashvili, Classes of hydrodynamic and magnetohydrodynamic turbulent decay, Phys. Rev. Lett. **118**, 055102 (2017).

[12] J. Reppin and R. Banerjee, Nonhelical turbulence and the inverse transfer of energy: a parameter study, Phys. Rev. E **96**, 053105 (2017).

[13] P. Bhat, M. Zhou, and N. F. Loureiro, Inverse energy transfer in decaying, three-dimensional, non-helical magnetic turbulence due to magnetic reconnection, Mon. Not. R. Astron. Soc. **501**, 3074 (2021).

[14] L. D. Landau and E. M. Lifshitz, *Fluid Mechanics* (Pergamon Press, 1959).

[15] P. A. Davidson, *Turbulence: an Introduction for Scientists and Engineers* (Oxford University Press, 2015).

[16] J. M. Wagstaff and R. Banerjee, Extragalactic magnetic fields unlikely generated at the electroweak phase transition, J. Cosmol. Astropart. Phys. **2016**, 002.

[17] F. Rincon, Dynamo theories, J. Plasma Phys. **85**, 205850401 (2019).

[18] D. N. Hosking and A. A. Schekochihin, Emergence of long-range correlations and thermal spectra in forced turbulence, arXiv:2202.00462 (2022).

[19] A. K. Galishnikova, M. W. Kunz, and A. A. Schekochihin, Tearing instability and current-sheet disruption in the turbulent dynamo, Phys. Rev. X **12**, 041027 (2022).

[20] J. Zrake, Inverse cascade of nonhelical magnetic turbulence in a relativistic fluid, Astrophys. J. Lett. **794**, L26 (2014).

[21] J. B. Taylor, Relaxation of toroidal plasma and generation of reverse magnetic fields, Phys. Rev. Lett. **33**, 1139 (1974).

[22] S. Servidio, W. H. Matthaeus, and P. Dmitruk, Depression of nonlinearity in decaying isotropic MHD turbulence, Phys. Rev. Lett. **100**, 095005 (2008).
